# Microencapsulated Bio-Based Rejuvenators for the Self-Healing of Bituminous Materials

**DOI:** 10.3390/ma13061446

**Published:** 2020-03-22

**Authors:** Jose Norambuena-Contreras, Luis E. Arteaga-Perez, Andrea Y. Guadarrama-Lezama, Rodrigo Briones, Juan F. Vivanco, Irene Gonzalez-Torre

**Affiliations:** 1 LabMAT, Department of Civil and Environmental Engineering, Universidad del Bío-Bío, Avenida Collao 1202, Concepción, Chile; irenegon@ubiobio.cl; 2 LPTC, Laboratory on Thermal and Catalytic Processes, Department of Wood Engineering, Universidad del Bío-Bío, Avenida Collao 1202, Concepción, Chile; larteaga@ubiobio.cl; 3 Facultad de Química, Universidad Autónoma del Estado de México, Paseo Colón esq. Paseo Tollocan s/n, Col. Residencial Colón 50120, Toluca 50000, Estado de México, Mexico; ayguadarramal@uaemex.mx; 4 CIPA, Centro de Investigación de Polímeros Avanzados, Avenida Collao 1202, Concepción, Chile; r.briones@cipachile.cl; 5 Facultad de Ingeniería y Ciencias, Universidad Adolfo Ibáñez, Viña del Mar 2562340, Chile; juan.vivanco@uai.cl

**Keywords:** asphalt, encapsulated rejuvenators, agricultural waste, bio-oil, self-healing efficiency

## Abstract

Asphalt self-healing by encapsulated rejuvenating agents is considered a revolutionary technology for the autonomic crack-healing of aged asphalt pavements. This paper aims to explore the use of Bio-Oil (BO) obtained from liquefied agricultural biomass waste as a bio-based encapsulated rejuvenating agent for self-healing of bituminous materials. Novel BO capsules were synthesized using two simple dripping methods through dropping funnel and syringe pump devices, where the BO agent was microencapsulated by external ionic gelation in a biopolymer matrix of sodium alginate. Size, surface aspect, and elemental composition of the BO capsules were characterized by optical and scanning electron microscopy and energy-dispersive X-ray spectroscopy. Thermal stability and chemical properties of BO capsules and their components were assessed through thermogravimetric analysis (TGA-DTG) and Fourier-Transform Infrared spectroscopy (FTIR-ATR). The mechanical behavior of the capsules was evaluated by compressive and low-load micro-indentation tests. The self-healing efficiency over time of BO as a rejuvenating agent in cracked bitumen samples was quantified by fluorescence microscopy. Main results showed that the BO capsules presented an adequate morphology for the asphalt self-healing application, with good thermal stability and physical-chemical properties. It was also proven that the BO can diffuse in the bitumen reducing the viscosity and consequently self-healing the open microcracks.

## 1. Introduction

**Figure 1:**
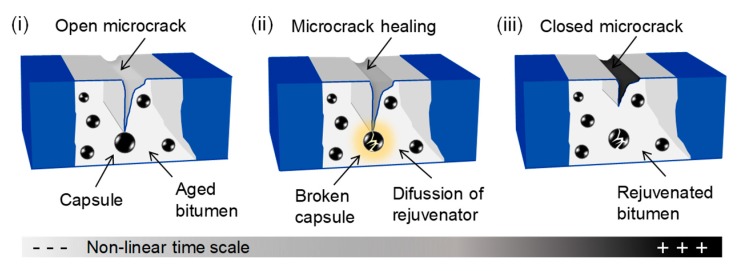
Concept of autonomic crack-healing in asphalt over time using encapsulated rejuvenators. Depending on their structure, capsules can be classified as either spherical polynuclear capsules or core-shell capsules [10], consisting of a defined core/cargo surrounded by a polymeric shell structure.

Bituminous materials are viscoelastic composites mainly used for road and airport pavement construction. These materials include asphalt mixtures, mastics and bituminous binders [1]. Amongst the bituminous materials, asphalt mixtures are the most used for asphalt pavement construction in the world. When asphalt pavements are exposed to years of mechanical and thermal stresses, as well as environmental effects (i.e., air oxidation, ultraviolet (UV) radiation, moisture), debonding at the interface between bitumen and mineral aggregates can occur, resulting in their cracking [2]. Cracking of bituminous materials mainly occurs due to the oxidation of the hydrocarbon compounds of bitumen [3], which is a dark thermoplastic material composed of a solid part called asphaltenes and a liquid part called maltenes (resins and oils) [4]. During the oxidative process, additional oxygen-containing polar functional groups promote clustering mechanisms, the content of asphaltenes increases, while maltenes decrease, and all these molecular-scale changes result in an increase of rigidity of the pavement and, consequently, its damage by cracking [5]. Hence, different approaches and technologies to promote crack closure at an early stage have been recently proposed, based on the development of self-healing bituminous materials [6]. Numerous advances on novel self-healing bituminous materials for sustainable pavements have been developed over recent years, turning this topic into an emerging field of study. Currently, two different approaches have mainly been used to promote asphalt self-healing thereby extending the service life of asphalt pavements: first, an approach to reduce the viscosity of bitumen by increasing its temperature through externally triggered heating via induction and microwave radiation [7] and, secondly, an autonomic approach by the action of encapsulated rejuvenating agents [8]. Rejuvenating agents consist of lubricating and extender oils with a high proportion of maltene constituents, which restore the asphaltenes/maltenes ratio in the aged bitumen (i.e., reduce the stiffness of the aged binder) [9]. The concept of self-healing in bituminous materials over non-linear time is shown in Figure 1. When damage occurs in an asphalt containing embedded capsules, open microcracks appear and eventually propagate until they reach and break a capsule releasing the contained rejuvenating agent, which reduces the viscosity of aged bitumen by diffusion, allowing it to flow through the cracks, sealing them autonomously.

Several authors have successfully synthesized and proven the use of numerous encapsulated rejuvenating agents, such as dense aromatic oil [11], waste cooking oil [12], and sunflower-cooking oil [13] with asphalt self-healing purposes. Encapsulation procedures, such as in-situ polymerization and ionic gelation, have mainly been used to produce core-shell and polynuclear (beads) encapsulated rejuvenators with size ranges of 5–153 µm and 2–7 mm, respectively [11,12,13,14,15,16]. Recently, bio-oil (BO) has been introduced by Zhang et al. [17] as a sustainable rejuvenating agent for aged asphalt binder. Bio-oil is a kind of renewable material which can be obtained from crops, cotton, straw, wood waste, animal manure and others through pyrolysis [18] or liquefaction processes [19]. In this context, Aguirre and co-workers [20] synthesised double-walled microcapsules with size 153 µm by in-situ polymerisation using polyurethane and urea-formaldehyde, containing a commercial BO product as the rejuvenating agent. Nonetheless, the petrochemical-based synthetic polymers used for the encapsulation process can produce a high environmental risk from the leaching of hazardous chemical compounds, making these capsules unsuitable for their use in asphalt pavements [21].

This paper aims to explore the use of BO obtained from liquefied agricultural biomass waste as a bio-based encapsulated rejuvenating agent for the self-healing of bituminous materials. To reach this aim, as a first stage of this research, BO capsules were synthesized using two simple dripping methods, where the BO agent was encapsulated by ionic gelation in a biopolymeric matrix of sodium alginate. This bio-based polymer was selected due to its large use in oil encapsulation and high capacity to form gel at low concentrations [14]. Finally, a comprehensive experimental characterization of the different BO capsules and their main results are discussed within the paper.

## 2. Materials and Methods

### 2.1. Materials

Biopolymeric BO capsules for asphalt self-healing were prepared in this study. The polymeric structure of the capsules was prepared of low-viscosity grade sodium alginate (viscosity at 20 °C 200–300 cP for 2% w/v solution) provided as a powder by Buchi (Flawil, Switzerland), and calcium chloride dihydrate (CaCl_2_·2H_2_O) provided in granular pellets with 70% purity by Winkler (Concepción, Chile). Bio-Oil (BO) obtained from agricultural biomass waste via liquefaction with density 1.25 g/cm^3^, viscosity at 20 °C 750 cP, and pH at 25 °C 2.0–2.5 was used as the bio-based rejuvenating agent for asphalt self-healing. Virgin bitumen with density 1.04 g/cm^3^ and penetration grade 80/100 mm at 25 °C was used to quantify the BO healing efficiency.

### 2.2. Preparation of Bio-Oil Rejuvenating Agent by Liquefaction Process

Bio-oil used as the rejuvenating agent for asphalt self-healing was obtained from agricultural biomass waste through a solvolysis liquefaction process described by Briones et al. [19]. Briefly, a mixture of PEG#400 and glycerol in proportion 50/50 w/w was used as the solvent in the liquefaction process. The liquefying solvent and a small amount of H_2_SO_4_ (3 wt.% of the solvent) as catalyst were added into a three-neck flask reactor (250 mL) equipped with a mechanical stirrer (Model OS40-Pro -LB Pro, Rocky Hill, CO, USA), temperature controller and condenser. When the temperature of the liquefying media reached 150 °C, a specific quantity of oven-dried biomass corn stover residues was added gradually. The mixture was continuously stirred during the liquefaction process (ca. 45 min) to obtain a homogeneously liquefied bio-oil product. After ending the reaction, the flask was cooled down to ambient temperature to stop the reaction and the liquefied products were collected for later application as encapsulated rejuvenator.

### 2.3. Preparation of Bio-Based Capsules Containing Liquefied Bio-Oil

**Figure 2:**
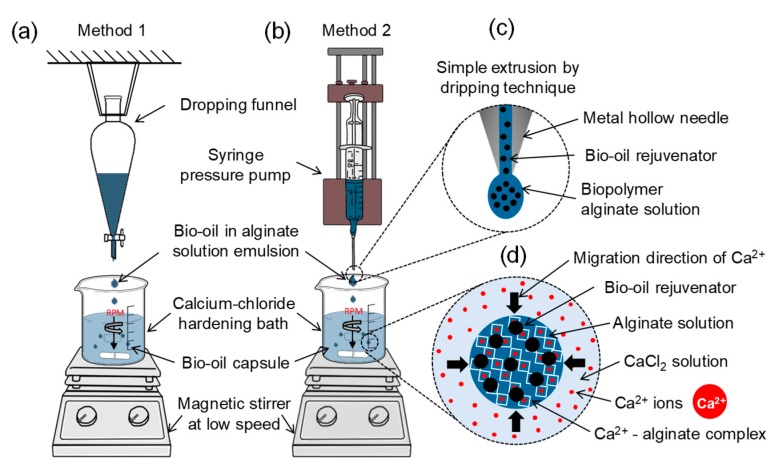
Preparation of the bio-based capsules containing bio-oil (BO). (**a**) Set-up of the dropping funnel method (M1); (**b**) set-up of the microfluidic pressure pump method (M2); (**c**) detail of BO encapsulation by simple extrusion through dripping technique using a syringe pressure pump, where the alginate emulsion is extruded through a metal hollow needle and added dropwise into a collecting/hardening bath where the biopolymer is cross-linked; (**d**) BO (BO) encapsulation in calcium alginate matrix via external gelation where Ca^2+^ ions migrate from the aqueous bath (CaCl_2_ solution) to the emulsion drop and, as a result, the alginate chains are progressively cross-linked forming irregular shaped or polynuclear BO capsules. (**c**,**d**) are based on Martins et al. [10].

Bio-oil capsules were prepared by the cross-linking of sodium alginate (C_6_H_7_O_6_Na) in the presence of Ca^2+^ ions through an external ionic gelation process [13], as shown in Figure 2. The preparation consisted of 5 steps, as follows: (1) sodium alginate was added to deionized water and mechanically stirred (Model OS40-Pro -LB Pro) at 700 rpm for 30 min, until its complete solution. After that, the solution was left to rest for about 30 min until all the bubbles disappeared; (2) rejuvenating BO was then added to the sodium alginate solution while mixing with the mechanical stirrer at 700 rpm until a stable emulsion was obtained; (3) at the same time, the hardening bath of calcium chloride (CaCl_2_) in deionized water was prepared, using a magnetic stirrer until complete solution; (4) capsules were formed by letting the water-alginate-rejuvenator emulsion drop into the calcium chloride hardening bath. During the capsule formation process, the hardening bath solution was gently agitated using a magnetic stirrer at 200 rpm. Capsules stayed in the solution for 30 min after the end of the encapsulation process; and (5) BO capsules were rinsed with deionized water and dried in an electric dryer for 24 h at 35 °C. Finally, the dried BO capsules were stored in a freezer at −18 °C to avoid the oxidation of the rejuvenator.

### 2.4. Concentrations and Encapsulation Procedures

To evaluate the effect of biopolymer concentrations and encapsulation methods on the properties of the capsules, two different sodium alginate concentrations (2% and 3% of weight by volume of water) and encapsulation methods were used for BO capsule preparation. For both concentrations, the BO/water volume ratio was 0.05. Two simple extrusion-dripping set-up methods by external gelation were used to prepare the BO capsules: a dropping funnel (M1), and a microfluidic pressure pump (M2). In both methods, the water-alginate-rejuvenator emulsion was dropped in the calcium chloride hardening bath. The dropping funnel method (see Figure 2a) constitutes a gravity-drip procedure, which mainly depends on the viscosity of the emulsion to form the capsules. In contrast, the pressure pump method (see Figure 2b) constitutes a controlled mechanism to produce capsules that pumps the emulsion through a metal hollow needle of 1.2 mm diameter at a pressure flow rate of 2 mL/min. Considering the variables involved in the study (2 methods and 2 concentrations) a total of 4 different types of BO capsules were prepared in this research, named as: M1–2%; M1–3%; M2–2%; and M2–3%.

### 2.5. Morphology, Encapsulation Efficiency, and Antioxidant Activity of Bio-Oil Capsules

The morphology of the BO capsules was characterized by their size, surface aspect and internal microstructure by means of Optical (Leica EZ4, Wetzlar, Germany) and Scanning Electron Microscopy (Hitachi SU 3500, Chiyoda, Tokyo, Japan), respectively. Additionally, the presence of elements in the surface of the BO capsules was evaluated by SEM through energy dispersive X-ray spectroscopy (EDX Bruker Quantax 100, Billerica, MA, USA) for semi-quantitative determinations. The relative density of the BO capsules was measured following method B of the ASTM D792-13 [22]. The encapsulation efficiency and antioxidant activity of the BO were quantified by chemical tests described by Guadarrama-Lezama et al. [23]. Thus, encapsulation efficiency of BO was defined as the ratio between the quantity of BO retained within the capsules and the total BO used to produce them. Additionally, antioxidant activity of the encapsulated BO was quantified by the 2,2′-azino-bis (3-ethylbenzothiazoline-6-sulphonic acid (ABTS) technique. Based on this method, the results of antioxidant activity of the BO were presented as µmol Trolox/mL.

### 2.6. Thermochemical Characterization of Bio-Oil Capsules and Their Components

Chemical structures of the BO capsules and BO were characterized by Attenuated-Total Reflection Infrared (ATR-IR) spectroscopy by direct transmittance in a single-reflection ATR system. Infrared spectra were recorded on a PerkinElmer Spectrum Two Fourier Transform Infrared Spectrometer (Waltham, Massachusetts, USA). Each spectrum was recorded over 20 scans in the range from 4000 to 400 cm^−1^, with a resolution of 2 cm^−1^. A thermogravimetric analysis (TGA-DGT) was carried out to evaluate the thermal stability of the BO capsules and their components. The thermal test was recorded on a TA Instrument TGA Q 50 thermogravimetric analyser (New Castle, Delaware, USA). The samples of ~5 mg were heated between ambient temperature and 600 °C at 10 °C/min. A constant nitrogen flow rate of 10 mL/min was used, ensuring an inert atmosphere during the pyrolysis process.

### 2.7. Mechanical Properties of Bio-Oil Capsules

**Figure 3:**
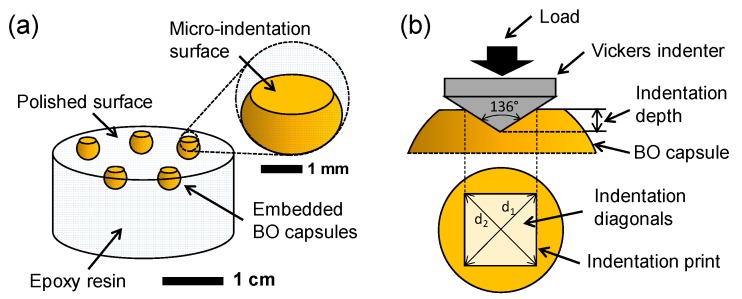
(**a**) Bio-oil (BO) capsules embedded in epoxy resin; (**b**) Vickers micro-indentation hardness testing. The Vickers micro-indenter is a square-based pyramidal-shape indenter made of diamond with an angle of 136° between opposite faces. A total of 40 tests were performed on BO capsules.

Compressive strength and micro-indentation hardness of the four different types of BO capsules were measured at 20 °C. Compressive strength of the BO capsules was measured following the recommendations of the ASTM D695-02 a [24]. For the tests, ten capsules of each type were randomly selected and pre-conditioned for 2 h at room temperature. The compressive strength was measured by uniaxial parallel plates compression testing on the 10 samples. To do that, individual capsules were loaded until failure at a loading rate of 0.2 mm/min using an Universal Testing Machine (Test Resources, Shakopee, Minnesota, USA) with a 5 kN load cell. Besides, a HV-1000A Microhardness Tester (Russell Fraser Sales Pty Ltd., Kirrawee, New South Wales, Australia) equipped with a Vickers indenter probe was used to perform the low-load micro-indentation tests on the BO capsules. To develop the tests, firstly, five BO capsules of each type were randomly selected and embedded in an epoxy resin mould (3 cm diameter and 2 cm height) and then polished until reaching the indentation surface (Figure 3a) to ensure a uniform surface to enable the quality of the micro-indentation process.

To carry out the micro-indentation tests, the indentation area was located on the centre of the BO capsules’ surface using the optical camera of the testing equipment. Micro-indentation tests consisted of applying a force of 50 gf for 10 s on the surface of each capsule, as shown in Figure 3b. After removing the force, the Vickers micro-indentation hardness (HV) of each BO capsule was calculated as follows:

(1) $$HV=\frac{2\cdot \sin\left(\frac{136{}^{\circ}}{2}\right)\cdot F}{d^{2}}\approx \frac{1.8544\cdot F}{d^{2}},$$

where *F* is the load in kgf, and *d* is the average diagonal length of the indentation print in mm.

### 2.8. Self-Healing Efficiency of Bio-Oil as Rejuvenating Agent: a Proof of Concept

The self-healing efficiency of the BO as a rejuvenating agent of asphalt was quantified in cracked bitumen samples by fluorescence microscopy. For the tests, thin film bitumen samples with dimensions 20 × 20 × 0.5 mm^3^ were prepared on a glass petri dish using masking tape and a metallic spatula. Then, hot bitumen test samples were conditioned for 2 h until they reached room temperature. After cooling of the samples, a 100 µm-width microcrack was made in the centre of the sample using a metallic cutting element. Then, using a hollow needle, a drop of BO ~2 mg was dropped on the cracked bitumen test sample to simulate the rejuvenator release into the open microcrack. The crack closure by the effect of BO diffusion over time was recorded periodically taking images with an inverted fluorescence microscope (ICOE IV 5100 FL, Ningbo, China) with phase contrast and a magnification up to 400×. Crack-width in the images was measured at six positions by image processing software ImageJ^®^ (Version 1.52u, NIH Wisconsin, Bethesda, Maryland, USA). The complete crack closing process was recorded over a maximum time of 120 min. Finally, the self-healing efficiency of the BO rejuvenating over time was quantified as follows:

(2) $$SHE_{ti}\left(\%\right)=\left(\frac{ICW_{0}-PCW_{ti}}{ICW_{0}}\right)\times 100,$$

where *SHE_ti_* is the healing efficiency at a time *t_i_* measured in %, *PCW_ti_* is the average partial crack-width at a time *t_i_* measured in µm, and ICW_0_ is the average initial crack-width measured in µm.

## 3. Results and Discussion

### 3.1. Morphology and Composition of the Bio-Oil Capsules

**Figure 4:**
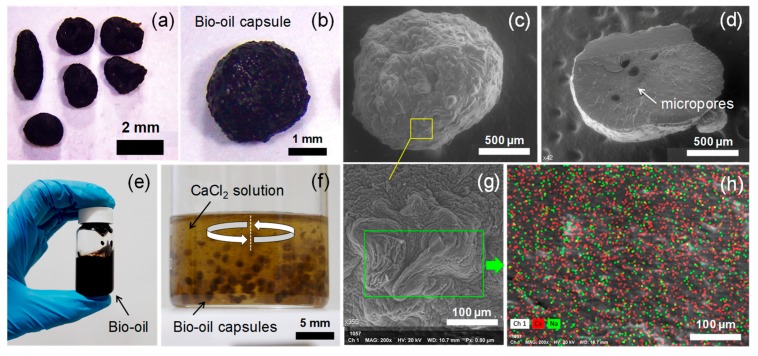
(**a**) Image of the different BO capsules produced as result of the alginate concentration and encapsulation method used; (**b**) individual spherical BO capsule; (**c**) SEM image of BO capsule; (**d**) SEM image of the cross-section of BO capsule; (**e**) liquid Bio-Oil (BO) obtained from liquefied agricultural biomass waste; (**f**) BO capsules in hardening bath of calcium chloride; (**g**) SEM detail image of surface structure on BO capsule; (**h**) SEM-EDS on the BO capsule surface.

Figure 4 presents the main results of the morphological and composition experimental characterization developed on the BO capsules. Results showed that BO capsules with different regular and irregular shaped size were obtained as result of the polymer concentration and encapsulation method used (Figure 4a). BO capsules with regular spherical size (Figure 4b) were produced majorly by method 2. SEM images of surface and cross-section of an individual BO capsule are shown in Figure 4c,d, respectively. From SEM images, it can be observed that the BO capsules showed a dense membrane in their surface, without polynuclear formation and internal multicavity “egg-box”. This result is not common considering the external ionic gelation method used [10], which suggests that BO chemically affects the encapsulating process increasing the cross-linking process of the alginate matrix and, as a result, endowing the capsules with a denser and more rigid membrane and polymer matrix. Regardless the limited microporous structure shown by BO capsules, they presented an encapsulation efficiency of BO of 78 ± 5%, for both concentrations and methods.

The black color of the BO capsules was given by the color of the BO product (Figure 4e), which also painted black the hardening bath of calcium chloride (Figure 4f) while the capsules were produced. In addition, SEM-EDS characterization proved that BO capsules reveal a rough texture on their surface (Figure 4g) with an element composition of Ca (56.43%) and Na (43.57%), as a result of the materials used for the synthesis of calcium alginate matrix structure in the presence of Ca^2+^ ions.

**Figure 5:**
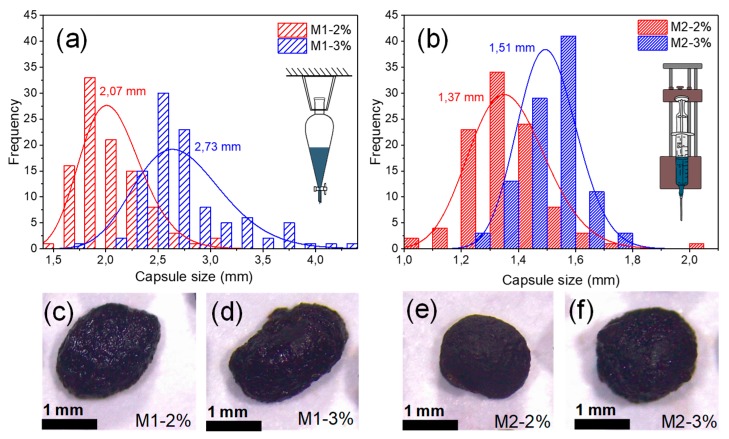
(**a**) Frequency histogram of the size of BO capsules with log-normal fitting produced by the dropping funnel method (M1); (**b**) frequency histogram of the size of BO capsules with log-normal fitting produced by the pressure pump method (M2); (**c**–**f**) examples of the produced BO capsules.

Furthermore, the distribution of the different BO capsule sizes is shown by the frequency histograms in Figure 5a,b, for capsules produced by method 1 and 2, respectively. This histogram proves that the BO capsule size can be fitted to the log-normal probability distribution (P-values 0.863 (M1–2%); 0.247 (M1–3%); 0.922 (M2–2%); and 0.676 (M2–3%) given by K-S test) used to model stochastic processes. Statistical size analysis of 100 individual BO capsules by each type registered an average size of 2.07 mm (SD = 0.32 mm), 2.73 mm (SD = 0.43 mm), 1.37 mm (SD = 0.14 mm) and 1.51 mm (SD = 0.10 mm) for capsules M1–2%, M1–3%, M2–2%, and M2–3%, respectively. These results proved that method 1 produced larger capsules than method 2 (51% and 82%, respectively) and that the increment of 1% of alginate (2% to 3%) increased the capsule size by 32% and 10% for methods 1 and 2, respectively. This result was due to the fact that the BO capsules produced by method 1 (Figure 5c,d) were more irregular compared with the capsules produced by the method 2 (Figure 5e,f).

**Figure 6:**
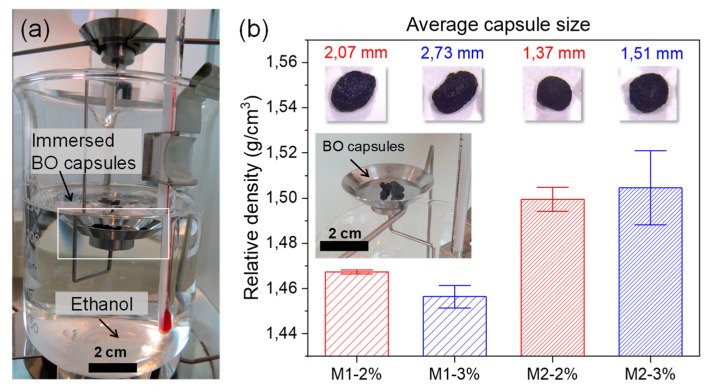
(**a**) Measurement of the relative density of the BO capsules with test method B of the ASTM D792-13 [22] used in the case of materials with densities that are very close to, or lighter than, water. In the image, the mass in air of a 100 mg sample BO of capsules was determined, and then it was immersed in ethanol with density 0.789 g/cm^3^, determining its apparent mass upon immersion and calculating its relative density; (**b**) average results of density of BO capsules with St. Dev. error bars.

Furthermore, the BO capsules’ size and their morphological micro-structure also affected their density. To prove it, Figure 6 shows the average results of the relative density measured on the different BO capsules. From these results, it can be concluded that BO capsules produced by method 2 (with smaller size and volume) presented a higher average relative density compared with those of method 1 and that the alginate concentration increase does not significantly affect their density value. The BO capsule type M2–3% (average size 1.51 mm) presents the highest value of density (1.505 g/cm^3^; SD = 0.023 g/cm^3^), while the capsule type M1–3% (average size 2.73 mm) registered the lowest density (1.467 g/cm^3^; SD = 0.001 g/cm^3^), as shown in Figure 6. These results suggest that, although the size (volume) of the BO capsules affected the density, the average density values for the capsules were very similar. Overall, the size and composition results of the BO capsules confirm that they can be added into asphalt matrix as an encapsulated rejuvenator with asphalt self-healing purposes.

### 3.2. Compressive Strength and Micro-Indentation Hardness of the Bio-Oil Capsules

**Figure 7:**
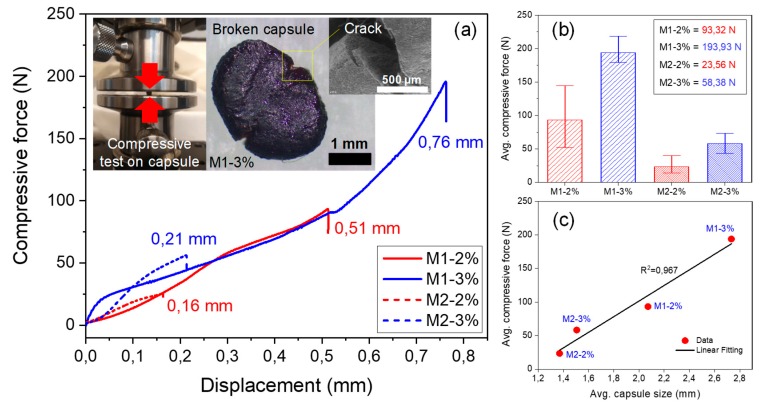
(**a**) Average curves of the compressive force versus displacement of the BO capsules tested following the recommendations of the ASTM D695-02a [24], including image details of the mechanical test, broken capsule, and crack type; (**b**) average results of maximum compressive force of capsules with St. Dev. error bars; (**c**) linear correlation between compressive force and capsule size.

Figure 7 summarizes the results of the compressive tests developed at 20 °C on the different BO capsules. Average curves of the compressive force versus displacement obtained of the capsules are shown in Figure 7a. It can be observed that the BO capsules presented an elastic-plastic mechanical behavior with moderate ductility and breakage in plastic deformation. As result, BO capsules M1–2% and M1–3% recorded the largest average compressive forces and displacements, with values of 93.32 N and 193.93 N at displacements of 0.51 mm and 0.76 mm, respectively. This result proves that the BO capsules increase their compressive force and displacement with the increase of the sodium alginate concentration (2 to 3%) and the encapsulation method used (M1 to M2, Figure 2).

Previous research [8] proved that the minimum compressive force of capsules required to resist the asphalt manufacturing processes was 10 N, approximately. Based on this study, the obtained compression results (Figure 7) suggest that all BO capsules tested at room temperature could: (1) survive the asphalt manufacturing process, and (2) resist high compressive strength until failure and, as a result, partially release the encapsulated BO by the effect of an external trigger (such as traffic loads). Norambuena-Contreras et al. [13] proved through compressive tests that the exposure of calcium alginate capsules to high temperatures (160 °C) degrades the polymeric matrix structure of the capsules making them more brittle, and hence less flexible to mechanical loads. However, the maximum force resisted by the BO capsules, as shown in Figure 7b, shows that most of the BO capsules (3 of 4 types) registered average compressive forces greater than those obtained by Norambuena-Contreras et al. [13] (50 N at 20 °C) for capsules containing sunflower oil as encapsulating rejuvenator. Thus, considering that the BO capsules have a greater mechanical performance than the calcium alginate capsules produced in [13], it can be concluded that the BO-capsules also can successfully resist the asphalt manufacturing process. However, this hypothesis should be checked as a second part of this study. Additionally, results shown in Figure 7b proved that the addition of a higher amount of sodium alginate (2 to 3%) to the composition of BO capsules helped to increase their compressive strength because the BO capsules present a denser internal structure, as shown in the SEM image of capsule cross-section in Figure 4d.

**Figure 8:**
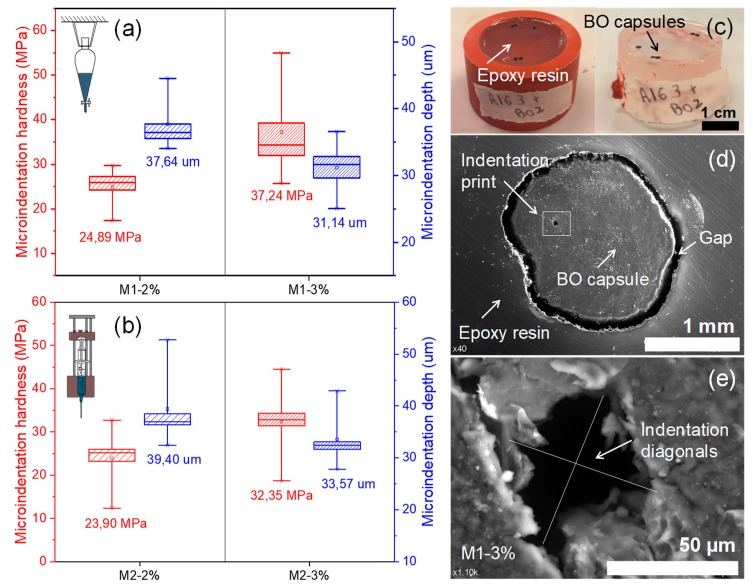
(**a**) Box plots of the results of Vickers micro-indentation hardness and depth for BO capsules produced by method 1; (**b**) box plots of the results of micro-indentation hardness and depth for BO capsules produced by method 2; (**c**) BO capsules embedded in a mould of epoxy resin; (**d**) SEM image of the BO capsule M1–3% taken 24 h after test; (**e**) SEM image detail of the micro-indentation print (i.e., indentation diagonals) in BO capsule M1–3% taken 24 h after test.

Furthermore, Figure 8 shows the results of Vickers micro-indentation hardness and depth registered for BO capsules produced by method 1 (Figure 8a) and method 2 (Figure 8b), respectively. From these box plots, it can be observed that sodium alginate concentration addition influenced the hardness and micro-indentation depth of capsules. Thus, BO capsules with 2% of alginate registered lower average hardness, M1–2% (24.89 MPa; SD = 4.67 MPa) and M2–2% (23.90 MPa; SD = 7.38 MPa), while capsules with 3% alginate registered the greatest average hardness, M1–3% (37.24 MPa; SD = 14.63 MPa) and M2–3% (32.35 MPa; SD = 9.22 MPa). Additionally, greater hardness results were obtained in a lower depth value. From Figure 8, it can be concluded that capsule M1–3% was the one that presented a lower depth (31.14 µm; SD = 11.48 µm) and, therefore, greater hardness.

This result coincides with the compressive results, where the BO capsule M1–3% recorded the greatest average maximum compressive force, as shown in Figure 7b. This result was due to the dense microstructure of the capsule, which allows it to have a high resistance to hardness and compression. However, this dense structure reduces its properties to release encapsulated BO. Figure 8c shows an example of the BO capsules embedded in epoxy resin and Figure 8d shows a cross-section of the capsule M1–3% after micro-indentation test. In this Figure, an individual micro-indentation print can be observed, which has been magnified in Figure 8e. The SEM characterization of the BO capsule test specimens, 24 h after carrying out the micro-indentation hardness test, suggested that the capsules revealed partial recovery of the deformation. For example, the average of indentation diagonals registered by the capsules M1–3% after test was close to 200 µm, while after 24 h, this value was reduced to 50 µm. This strain recovery phenomenon shown by the BO capsules on the indentation tests was due to their elastic-plastic mechanical behavior previously proved by the compressive test, as shown in Figure 7a. In summary, the micro-indentation hardness results obtained (Figure 8) suggest that all BO capsules tested at room temperature could resist the indentation loads produced by the aggregates during the asphalt manufacturing process; hence, they could survive to partially release the encapsulated BO in the asphalt matrix by the effect of an external force trigger.

### 3.3. Chemical and Thermal Properties of the BO Capsules and Their Components

**Figure 9:**
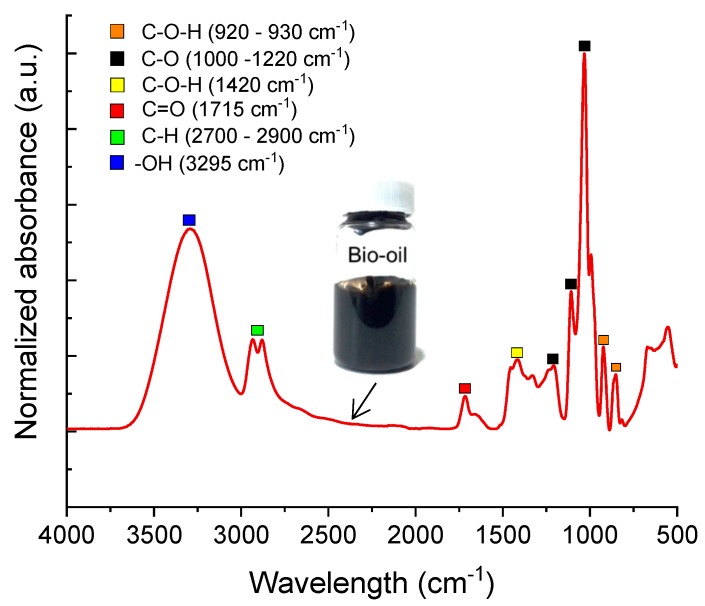
Normalized infrared spectra of BO obtained from liquefaction of corn stover residues. The assignment of spectral bands was carried out based on that previously reported by Lievens et al. [25] and Stuart [26] on BO and oxygen-containing compounds.

The BO sample was characterized by FTIR-ATR with the aim of identifying specific functional groups which could lead to an antioxidant activity of the BO and, as result, to a better performance as rejuvenating agent of aged asphalt binder. Figure 9 shows the normalized infrared spectra of BO obtained from the liquefaction process of corn stover residues. The assignment of spectral bands in the FTIR spectra was carried out based on that previously reported by Lievens et al. [25] and Stuart [26], regarding BO and oxygen-containing compounds. According to the results shown in Figure 9, the wavenumbers of substantial functional groups in BO are concentrated between 920 and 1720 cm^−1^, indicating the presence of species containing C–O–H, C–O and C=O bonds. These chemical species, usually acids, aldehydes, ketones and phenolics, derive from the deconstruction of cellulose and lignin during the liquefaction process, as has been widely reported [19]. Indeed, the absorption bands recorded between 1000 and 1220 cm^−1^ are associated with the C–O stretching in alcohols, glycols and phenolics. In particular, the lignin-derived phenolics have been identified as species with the potential to avoid the action of free radicals, thus with antioxidant properties [27].

Moreover, the absorption band at 3295 cm^−1^ could be associated with the O–H stretching in water and carboxylic acids. The latter is also confirmed by both C–O–H in-plane and C–O–H out-of-plane bending signals at 1420 cm^−1^ and 930 cm^−1^, respectively. The carboxylic acids have been identified as antioxidant compounds and one of the most important functionalities within the BO used [19]. Those signals found between 2700 and 2900 cm^−1^ are attributed to the C–H stretching in aliphatic groups (e.g., in aldehydes and ketones). The ketones are also responsible for increasing antioxidant activity and, in some applications, their antioxidant effect can conjugate with that of phenolics. In fact, the antioxidant activity of the BO measured by the 2,2′-azino-bis (3-ethylbenzothiazoline-6-sulphonic acid-ABTS) technique was 126.30 ± 2.7 μmol Trolox/mL, which is considerably greater than that reported for the antioxidant activity of sunflower oils (23.2 ± 1.1 μmol Trolox/mL) reported by Guadarrama-Lezama et al. [23]. Sunflower oil was successfully encapsulated in calcium alginate (CaAlg) capsules as a rejuvenating material with asphalt self-healing purposes.

In this context, Norambuena-Contreras et al. [13] proved that the sunflower oil released from the CaAlg capsules significantly increases the self-healing capability of the dense asphalt mixture [14] and Stone Mastic Asphalt [15]. However, when the CaAlg capsules are exposed to high temperatures for long pre-heating periods, they can suffer peroxidation of the encapsulated sunflower oil [13]. Considering the FTIR-ATR evidence, it can be concluded that the BO obtained by liquefaction of corn stover residues has strong antioxidant properties provided by carboxylic acids, phenolics and, to less extent, by ketone functionalities. So, this liquid has the potential to be used for asphalt self-healing applications. However, for future encapsulation applications by means of biopolymers, interactions between the BO and capsule materials should be carefully analyzed. In this study it was proven that the interaction of carboxylic acids, CaCl_2_ and alginate could lead to a cross-linked structure of the BO capsule and even to the erosion of its surface, as shown in the SEM image in Figure 4g, which may decrease the efficiency in releasing the BO during the asphalt healing process [28].

**Figure 10:**
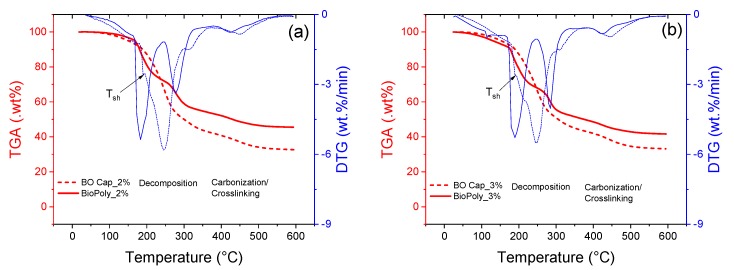
TGA and DTG results of the biopolymer matrix (BioPoly) and BO capsules (BO Cap) for concentrations of (**a**) 2% of alginate; and (**b**) 3% of alginate. T_sh_ is the DTG shoulder temperature.

Furthermore, thermogravimetry (TGA) and derivative thermogravimetry (DTG) profiles of the biopolymer (BioPoly) containing 2% and 3% of alginate are shown in Figure 10 along with the capsules (BO Cap) prepared from those biopolymers and containing the BO. The thermal stability of the biopolymer (BioPoly) used for the preparation of the capsules was confirmed by the TGA curves in Figure 10. From these figures, it can be observed that the decomposition at 160 °C—temperature of asphalt mixture preparation—was below 10% for both BioPoly_2% (Figure 10a) and BioPoly_3% (Figure 10b), respectively. Two major DTG peaks, corresponding to 10–12% and 36–40% wt. loss, were identified for the BioPoly. The first peak around 185 °C is associated with dehydration reactions in the alginate; while the second one, at 278 °C, corresponds to the degradation of the CaCO_3_.

Likewise, the weight loss below 300 °C is caused by the loss of hydroxyl groups (–OH) in the alginate, and above this temperature decarboxylation reactions take place forming CO_2_ as the main product [29]. When BO is confined into the CaAlg capsules (BO Cap_2% and BO Cap_3%) the TGA profiles of the BioPoly and that of the capsules containing the BO are very similar until 175 °C. Above 175 °C, the decomposition of the BO starts showing a DTG shoulder at Tsh = 190 °C, which could be attributed to the breakage of C‒H and C–O–H in lighter molecules, such as carboxylic acids, and, to some extent, to the decomposition of the alginate itself. Then, a maximum peak is found around 250 °C corresponding to the overlapping of the BO capsule decomposition and to the pyrolysis of aldehydes, phenolics and ketones contained in the BO (based on Figure 9). Those peaks found above 400 °C correspond to the carbonization of the alginate matrix via cross-linking reactions.

Overall, TGA results of BO capsules and their components are in line with those found in the BO spectrum FTIR and suggest that the encapsulation process of BO as rejuvenating agent leads to a thermally stable material with the potential to be used for asphalt self-healing applications.

### 3.4. Self-Healing Efficiency of the Bio-Oil as Encapsulated Rejuvenator

**Figure 11:**
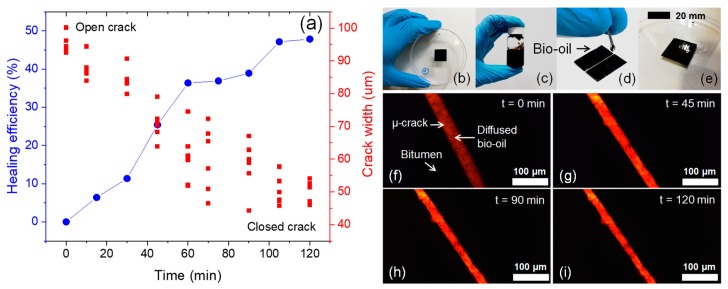
(**a**) Results of healing efficiency and µ-crack with over time; (**b–e**) Procedure to measure the healing efficiency of BO on film bitumen test samples of 20 × 20 × 0.5 mm; (**f–i**) Fluorescence microscopy images on closed µ-crack by the effect of BO diffused in bitumen over time.

The self-healing capability of the BO as rejuvenating agent for asphalt was quantified in this study by means of fluorescence microscopy. Figure 11 presents the results of healing efficiency of BO as a proof of concept measured over time. From this Figure, it can be observed that BO obtained from the liquefaction of corn stover residues has the capability to close the µ-crack in the bitumen by effect of the diffusion of BO in bitumen. In Figure 11a, it can be seen that BO has a healing efficiency close to 50% at a time of 120 min of evaluation. Likewise, the µcrack-width showed a linear trend with the healing time. The healing process occurs because the bio-rejuvenator (i.e., BO) increases the content of viscous components of asphalts and decreases the viscosity of bitumen, allowing it to flow over time healing its open crack. In short, BO can dissolve the bitumen on both sides of the crack. Likewise, fluorescence images have allowed the measurement of the crack-closing over time because the diffusion of bituminous molecules caused by Brownian motion [30] was accelerated by the BO.

The self-healing capability results of BO in virgin bitumen are promising and future application in aged asphalt samples should be evaluated. Zhang et al. [17] reported that the addition of bio-rejuvenator from biomass waste decreases the activation energy of aged asphalt binder and increases the temperature susceptibility and fluidity of aged asphalt binder, with the potential to rejuvenate it.

Previous studies by Aguirre et al. [20] proved that encapsulated commercial BO can be used for asphalt self-healing. So, encapsulated BO obtained from liquefaction of corn stover residues can be potentially used as a sustainable rejuvenator for aged asphalts in order to promote their µcrack-healing. Nevertheless, future research using different amounts of BO capsules should be carried out to prove this effect in bituminous materials (i.e., including asphalt mixtures, asphalt mastics and bituminous binders) along all different length scales.

## 4. Conclusions and Future Research

This paper explored the use of BO obtained from liquefied agricultural biomass waste as a potential bio-based encapsulated rejuvenating agent for asphalt self-healing. Based on the results, the following conclusions have been obtained.

Bio-Oil (BO) capsules with different size (regular and irregular shaped size) and with a BO encapsulation efficiency of 78% were obtained as result of two different polymer concentrations (2% and 3%) and using two different encapsulating dripping methods: a dropping funnel (M1) and a microfluidic pressure pump (M2). Encapsulating method 1 produced BO capsules with greater size (average range size 2.07–2.73 mm) than encapsulating method 2 (average range size 1.37–1.51 mm). In addition, it was proven that the increase of 1% of alginate (2% to 3%) increased the capsule size.

BO capsules produced by method 1 were more irregular compared with the capsules produced by method 2. SEM characterization on capsule samples showed that the BO capsules presented a dense membrane on their surface, without polynuclear formation and internal multicavity.

Likewise, it was proven that BO capsule size and their micro-structure affected their density. BO capsules M2–3% (average size 1.51 mm) presented the highest value of density (1.505 g/cm^3^), while capsules M1–3% (average size 2.73 mm) registered the lowest density (1.467 g/cm^3^).

BO capsules presented an elastic-plastic mechanical behavior with moderate ductility and breakage in plastic deformation. Results of compressive characterization proved that the BO capsules increased their compressive force and displacement with the increase of sodium alginate concentration (2 to 3%) and the encapsulation method used (M1 to M2).

Compression results obtained suggest that all BO capsules tested at room temperature could survive the asphalt manufacturing process and resist high compressive strength until failure and, as a result, partially release the encapsulated BO by the effect of an external trigger (such as traffic loads).

Additionally, it was proven that sodium alginate concentration addition influenced the hardness and depth of BO capsules. BO capsules with 2% of alginate registered the lowest average hardness, while capsules with 3% alginate registered the greatest average hardness.

Moreover, greater hardness results were obtained at a lower depth value. It was concluded that capsule M1–3% was the one that presented a lower depth and hence a greater hardness. This result coincides with the compressive results. In short, micro-indentation hardness results obtained suggest that all BO capsules tested at room temperature could resist the indentation loads produced by the aggregates during the asphalt manufacturing process, and therefore, survive for partial release of the encapsulated BO in the asphalt matrix by effect of an external force trigger.

Based on FTIR-ATR results, it was concluded that the BO obtained by liquefaction of corn stover residues has strong antioxidant properties (antioxidant activity 126.30 μmol Trolox/mL). So, this liquid has the potential to be used for asphalt self-healing applications.

Overall, TGA results of BO capsules and their components were in accordance with the BO spectrum FTIR and allow the suggestion that the encapsulation process of BO as a rejuvenating agent leads to a thermally stable material with the potential to be used for asphalt self-healing applications.

It was proven that BO has the capability to close the µ-crack in the bitumen by the effect of the diffusion of BO in bitumen, achieving a healing efficiency close to 50% at a time of 120 min of evaluation. Self-healing capability results of BO in virgin bitumen are promising for evaluation of its future application in aged asphalt sample and, hence, encapsulated BO can be potentially used as a sustainable rejuvenator for aged asphalts with the aim of promoting their µcrack-healing.

Future research to evaluate in more detail the chemical interactions between the BO and capsule materials should be developed because these interactions could lead to a cross-linked structure of the BO capsule and even to the erosion of its surface, which may decrease the efficiency in releasing the BO rejuvenating agent during the asphalt healing process. To avoid this phenomenon, this study proposes a future research, albeit with the use of the coextrusion dripping technique to encapsulate the BO agent. Additionally, based on the limitations of this study, as future research the authors suggest carrying out several tests to 1) evaluate the rejuvenating effect of the BO on short-term and long-term aged binders by FTIR-ATR tests; 2) evaluate the spatial distribution of the BO capsules and their integrity inside the asphalt mixtures by X-ray computed tomography; and 3) quantify by tests the effect of the mixing order and the aging time on the mechanical stability and self-healing properties of asphalt mixtures with, and without, BO capsules.
